# Emotional management and clinical communication among nursing students: a single institution experience

**DOI:** 10.3389/fpsyt.2024.1327629

**Published:** 2024-11-04

**Authors:** Meiqiong Yang, Pingzhen Lin, Limian Zheng, Biyu Wu

**Affiliations:** Department of Nursing, Quanzhou First Hospital, Quanzhou, Fujian, China

**Keywords:** care, communication, education, emotion, management

## Abstract

**Background:**

The development of emotional and clinical communication is crucial for being a nurse. The aim of this study is to evaluate the influencing factors of emotional management and clinical communication competencies among nursing students, to inform strategies for enhancing the management and nursing care practices.

**Methods:**

This study was a cross-sectional survey. The study period was from December 1 to December 31, 2022, during which nursing students were the population of the survey. The assessments of the emotional management and clinical communication competencies of the nursing students were conducted using the validated Emotion Management Ability Questionnaire and the Clinical Communication Ability Scale, respectively.

**Results:**

A total of 356 nursing students were investigated in this study. The nursing students had moderate ability of emotional management and clinical communication. The emotional management ability was correlated with the clinical communication ability of nursing students (all p<0.05). The results of multivariate regression analysis indicated that gender, educational level and home place were the independent influencing factors on the emotional management ability in the nursing students (all p<0.05). Family structure, internship time and personality were the independent influencing factors for the clinical communication ability among the nursing students (all p<0.05).

**Conclusions:**

The competencies in emotional management and clinical communication among clinical nursing students warrant enhancement. It is imperative to implement tailored educational and training programs to optimize the development and performance of nursing students in their clinical training.

## Background

Within the framework of Chinese nursing education, students must initially engage in a rigorous 2 to 3 year academic program focused on nursing theory. Subsequently, they are required to undertake a clinical internship, ranging from six months to one year in duration. It is only after the successful completion of these educational and practical components that they become eligible to participate in the nursing qualification examination (1, 2). Nursing students are engaged in the process of completing nursing education and clinical practice, preparing to attain their nursing licensure (3). It denotes the initial phase of the nursing career trajectory, signifying the commencement of a professional nursing career. In the face of busy nursing work and complex interpersonal relationships, nursing students need take effective measures to control their emotions (4). Some studies have pointed out that targeted emotional management of nurses can not only effectively improve the ability of nurses to cope with stress, but also help to improve the effect of clinical nursing practice (5–7). In the process of social development, we realize that people need more and more emotional management. Emotional management can regulate not only the individual level, but also the emotion of the group (8). Therefore, in addition to improving the external environmental factors affecting nursing students, managers should also consider how to improve nurses’ emotional management ability, so that nursing students can effectively manage their emotions and provide safe and effective clinical nursing (9).

Clinical communication ability refers to the ability of nurses to exchange information with patients and their families or other medical personnel in order to promote the recovery, and maintenance of patients’ health needs under the current patient-centered medical model (10). Many nursing experts believe that nurses’ clinical communication ability can fully reflect the occupational price of nurses, and that clinical communication ability should be defined as one of the core qualities of nursing professional ability (11, 12). Some even think that compared with simple skill operation, communication between nurses and patients, colleagues, superiors and subordinates is more important in daily nursing work (13). Previous study has shown that the prevalence of complaints regarding nursing services is predominantly attributed to inadequate information exchange and communication barriers between nursing staff and patients (14). Conversely, the facilitation of seamless information exchange is identified as a pivotal determinant in enhancing patient satisfaction. Consequently, there is an imperative need for research that examines the proficiency levels of clinical communication among nursing students and the factors that influence these competencies (15). Therefore, the aim of this survey is to provide an evaluation of the factors related to the emotional management and clinical communication ability of nursing students, to inform and enhance clinical nursing education and management practices.

## Methods

### Study design

This study was a cross-sectional survey design.

### Ethical statements

This study has undergone a thorough ethical review and has been duly approved by the hospital’s ethics committee (approval number 20210018-2a). Additionally, we have ensured that written informed consent was obtained from all participating nursing students prior to their inclusion in the study.

### Sample size consideration

In accordance with the methodological guidelines outlined in a prior report (17), it is widely recommended that the optimal sample size for a questionnaire-based study should be at least 20 times the number of variables under analysis. For this investigation, which utilized a scale comprising seven distinct variables, the requisite sample size was meticulously ascertained. Considering an expected attrition rate of 20% during the survey dissemination and collection phase, and to maintain statistical rigor, the calculated sample size was determined to be at least (7 *20*(1 + 20%) = 168) participants. Consequently, the study protocol mandated the enrollment of a minimum of 168 nursing students to comply with methodological standards and to ensure the reliability and validity of the findings.

### Study population

In this study, a questionnaire survey was conducted among nursing students in our hospital from December 1 to 31, 2022. These nursing students were from various affiliated partner colleges and all needed to undergo 6 to 12 months of nursing practice internship at our hospital. The inclusion criteria of nursing students in this study were as follows: Nursing interns who had completed at least one month of internship at our hospital; the nursing students signed the informed consent and voluntary participated in this study.

### Instrument

We compiled the general information questionnaire of nursing students, and we collected following information including gender, age, educational level, home address, personality, internship time and family structure.

#### Emotion management ability questionnaire

The questionnaire (16) included five factors: emotional expression ability, emotional awareness ability, emotional regulation ability, emotional understanding ability and emotional discrimination ability. The reliability index of five grades was adopted. The homogeneity reliability of each factor was 0.737 ~ 0.788, and the total reliability of the questionnaire was 0.829. The split-half reliability of each factor was 0.535 ~ 0.765, and the split-half reliability of the total questionnaire was 0.830. The correlation coefficient between each dimension was 0.411 ~ 0.619, and the correlation coefficient between each dimension and the total scale was 0.640 ~ 0.795. Many previous studies (17–19) have shown that the questionnaire had good reliability and validity and could be used as a tool to measure the ability of emotional management.

#### Clinical communication ability scale

The scale (20) included 6 dimensions with 28 items: ability of establishing a harmonious relationship, ability of listening keenly, ability of identifying patient’s problems, ability of verifying the feeling, ability of joint participation, ability of transmitting effective information. The content validity was 0.84, and factor analysis showed that 6 factors explained 53.77% of the total variance of the project. The total Cronbach’ s α coefficient was 0.84,0.67-0.80 for each dimension, 0.70 for split-half reliability coefficient, 0.56-0.81 for each factor split-half reliability coefficient, and 0.84 for test-retest correlation coefficient of the total scale. The test-retest correlation coefficients of each factor were 0.61 ~ 0.85. The scale had good validity and could be used to measure the clinical communication ability of nursing students (21, 22).

### Data collection

In this study, the questionnaire was filled out anonymously, and after consultation with the nursing management departments of various hospitals, the questionnaire was distributed. Before issuing the questionnaire, the researcher explained to the subjects to help nursing students understand and explain the purpose and matters needing attention of this study, so as to ensure the standardization and accuracy of the questionnaire. We provided unified guidance to the subjects before filling out the questionnaire and explain the matters needing attention in filling in the questionnaire. Nursing students were required to avoid discussing and talking with each other when filling out the questionnaire so as to reduce the response bias. The paper version of the questionnaire was collected on the spot and we used a box for the comes back to the questionnaire.

### Data analysis

This study used SPSS 23.0 software for statistical analysis. The measurement data were expressed by (mean ± standard deviation), and the counting data were analyzed by cases (percentage). T-test or analysis of variance was used to compare the scores of nursing students with different demographic characteristics. The relationship of emotional management and clinical communication ability of nursing students was analyzed by Pearson correlation. Multiple stratified regression analysis was used to explore the related factors of nursing students, and the difference was statistically significant when p< 0.05.

## Results

A total of 356 nursing students were included in this study. As presented in **Table 1**, of the included nursing students, 94.94% were female, with an average age of 21.08 years.

**Table 1:** The demographics of participants.

Characteristic	Cases(n=356)	Percentage
Gender		
Female	338	94.94%
Male	18	5.06%
Age(y)		
<20	62	17.42%
20~25	284	79.76%
>25	10	2.82%
Education level		
Bachelor degree	89	25.00%
Junior college degree	225	63.20%
Technical secondary school degree	42	11.80%
Home place		
Urban area	139	39.04%
Rural area	217	60.96%
Family structure		
Two parents	324	91.01%
Single parent	30	8.43%
No parent	2	0.56%
Internship time(months)		
<3	103	28.93%
3~6	121	33.99%
>6	132	37.08%
Personality		
Introverted	110	30.90%
Extroverted	246	69.10%

As presented in **Table 2**, the total score of emotional management of included nursing students was 82.96± 11.44, the total score of clinical communication of included nursing students was 80.39± 8.12, indicating that the nursing students had moderate ability of emotional management and clinical communication.

**Table 2:** The scores of emotional management and clinical communication ability of nursing students.

Item	Ability	Average score
Emotional management	Emotional expression ability	22.96 ± 4.70
	Emotion regulation ability	22.65± 5.03
	Emotional awareness ability	15.52± 4.16
	Emotional discrimination ability	11.69± 3.93
	Emotional understanding ability	11.27± 3.05
	Total score	82.96± 11.44
Clinical communication	Ability of establishing a harmonious relationship	16.56± 3.01
	Ability of listening keenly	15.84± 3.22
	Ability of identifying patient’s problems	15.77± 2.97
	Ability of verifying the feeling	14.60± 3.07
	Ability of joint participation	9.81± 2.15
	Ability of transmiting effective information	7.94± 2.01
	Total score	80.39± 8.12

As indicated in **Table 3**, the emotional management ability was correlated with the clinical communication ability of nursing students (all p<0.05).

**Table 3:** Correlation analysis of emotional management and clinical communication ability of nursing students.

Item	Ability of establishing a harmonious relationship	Ability of listening keenly	Ability of identifying patient’s problems	Ability of verifying the feeling	Ability of joint participation	Ability of transmitting effective information	Total score
Emotional expression ability	0.314^*^	0.435^*^	0.283^*^	0.396^*^	0.285^*^	-0.077	0.378^*^
Emotion regulation ability	0.299^*^	0.367^*^	0.355^*^	0.409^*^	0.092	-0.101	0.395^*^
Emotional awareness ability	0.275^*^	0.443^*^	0.489^*^	0.477^*^	0.108	0.032	0.405^*^
Emotional discrimination ability	0.308^*^	0.389^*^	0.401^*^	0.343^*^	0.065	-0.014	0.319^*^
Emotional understanding ability	0.284^*^	0.424^*^	0.376^*^	0.407^*^	0.041	-0.048	0.244^*^
Total score	0.422^*^	0.337^*^	0.345^*^	0.338^*^	0.079	-0.066	0.486^*^

^*^p<0.05.

As shown in **Table 4**, significant factors associated with emotional management are gender, educational level and home location(all P<0.05). Significant factors associated with clinical communication are family structure, internship time and personality(all P<0.05).

**Table 4:** Univariate analysis on the scores of emotional management and clinical communication ability of nursing students.

Characteristic	Cases(n=356)	Emotional management			Clinical communication		
		Score	t/F	p	Score	t/F	p
Gender			11.842	0.003		10.04	0.119
Female	338	83.10 ± 18.27			80.41 ± 18.02		
Male	18	74.94 ± 16.01			80.13 ± 19.25		
Age(y)			12.045	0.059		11.497	0.101
<20	62	78.24 ± 17.86			80.11 ± 18.06		
20~25	284	82.20 ± 18.56			80.02 ± 18.84		
>25	10	83.14 ± 17.04			80.39 ± 19.05		
Education level			12.117	0.044		12.004	0.116
Bachelor degree	89	83.03 ± 18.37			82.89 ± 18.14		
Junior college degree	225	82.17 ± 19.54			80.25 ± 19.06		
Technical secondary school degree	42	80.22 ± 18.85			79.87 ± 18.33		
Home place			11.842	0.001		11.878	0.063
Urban area	139	84.69 ± 20.14			81.56 ± 19.64		
Rural area	217	80.77 ± 19.31			79.77 ± 19.09		
Family structure			11.806	0.195		12.041	0.017
Two parents	324	83.12 ± 19.05			80.85 ± 20.15		
Single parent	30	80.55 ± 20.13			77.03 ± 19.31		
No parent	2	78.19 ± 19.58			75.38 ± 20.07		
Internship time(months)			12.471	0.112		11.899	0.024
<3	103	80.29 ± 20.08			77.31 ± 18.74		
3~6	121	81.74 ± 19.33			79.09 ± 20.55		
>6	132	83.02 ± 18.98			81.84 ± 19.16		
Personality			11.445	0.103		12.084	0.008
Introverted	110	81.09 ± 19.27			82.17 ± 19.05		
Extroverted	246	83.17 ± 19.44			80.25 ± 19.38		

As indicated in **Table 5**, gender, educational level and home place were the independent influencing factors on the emotional management ability in the nursing students (all p<0.05). Family structure, internship time and personality were the independent influencing factors for the clinical communication ability in the nursing students (all p<0.05).

**Table 5:** Multivariate regression analysis on influencing factors of emotional management and clinical communication ability of nursing students.

Scores	Variables	Standard error	β	t	P
Emotional management	Gender	0.506	0.403	6.181	0.004
	Education level	0.443	0.188	3.965	0.012
	Home place	0.345	0.128	2.015	0.009
Clinical communication	Family structure	0.588	0.184	2.086	0.013
	Internship time(months)	0.392	0.204	2.412	0.021
	Personality	0.417	0.211	2.875	0.017

**Table 5** Multivariate regression analysis on influencing factors of emotional management and clinical communication ability of nursing students

## Discussions

Appreciating the current dynamics and determinants of emotional management and clinical communication among nursing students is crucial for refining and augmenting clinical nursing education and administrative strategies. The results of this study show that the emotion management ability of nursing students needs to improved. Among the factors of emotional management ability, the highest score is emotional performance ability, followed by emotional regulation ability, emotional awareness ability, emotional discrimination ability, emotional understanding ability. The results of this study are consistent with those of previous surveys (23, 24). The observed emotional lability among nursing students, particularly those recently engaged in clinical practice, is a noteworthy phenomenon. This instability, coupled with the observed challenges in emotional regulation, could be a significant factor influencing their performance. Our survey data reveal that the emotional comprehension scores of nursing students are significantly lower than expected. This discrepancy may be attributed to several factors, including the limited duration of their clinical exposure, which potentially impedes their capacity to fully empathize with patients. Furthermore, the age of the nursing students could be a contributing factor, suggesting a need for targeted educational interventions to enhance their emotional intelligence and clinical empathy skills.

In China, the training process for nurses encompasses foundational nursing knowledge education, professional skills training, and clinical nursing practice. Only after passing rigorous assessments can nurses obtain their professional qualifications. At present, nursing students who are in the stage of clinical nursing practice have already received training in clinical communication and nursing psychology within an academic setting, thereby possessing a certain level of theoretical knowledge (25). The purpose of this phase of training is to integrate theoretical knowledge with clinical practice, thereby enhancing the professional skills and clinical nursing capabilities of the nursing students. The scores in emotional management ability exhibit a statistically significant variance between female and male nursing students in this study, potentially attributable to the inherent personality disparities between the genders. Some studies (26, 27) have found that males tend to exhibit a higher propensity for impulsive actions compared to females, a trait that could be linked to the distinct physiological attributes of the sexes. This predisposition often results in a diminished capacity for rational emotional regulation among males. In contrast, females are generally more inclined to display tolerance and resilience. Faced with emotional distress, males frequently opt to endure their discomfort in solitude, whereas females are more likely to seek solace and support by confiding in their peers or family members, thereby finding an avenue to alleviate their emotional strain through external assistance (28, 29). Besides, the emotion management ability of nursing students whose home address is urban is better than that in rural areas, which may be due to the family environment, family education and democratic rearing style of nursing students growing up in urban areas, and their parents may have a high degree of education (30, 31). At the same time, the urban living environment makes nursing students have more reading experience, more social experience, and relatively strong emotional management and control ability. The nursing students growing up in rural areas may form an authoritative or laissez-faire education model because of the lack of communication between parents and their children or the improper way of communication (30, 32). Studies (33, 34) have shown that children from democratic families have the strongest ability to manage stress and emotion, while children from laissez-faire families have weaker ability to manage stress and emotion. Therefore, when cultivating the emotional management ability of nursing students, nursing educators should focus on training nursing students and narrow the gap of emotional management ability between urban and rural nursing students.

This survey shows that the clinical communication ability of nursing students is average. The score of establishing harmonious relationship among nursing students is the highest, indicating that nursing students pay more attention to communication with patients, which may be related to the increasingly tense relationship between nurses and patients (35, 36). The nursing students whose family structure is two parents have the best clinical communication ability, which may be because the family relationship of two-parent families is generally harmonious (37). The family relationship is harmonious, and the relationship between nursing students and their parents is relatively good. Regular communication with their parents can predict their strong clinical communication ability, which is consistent with the results of previous studies (38, 39). The nursing students of two-parent families are relatively perfect both in personality and spiritually, and the good family relationship provides a good environment for the growth of nursing students (40). Besides, it can cultivate nursing students’ ability to communicate with others in verbal and non-verbal forms (41, 42). Additionally, the longer the internship, the better the clinical communication ability of the nursing students, and the longer the internship time, the more opportunities for interpersonal interaction and communication (43). At the same time, the accumulation of professional knowledge and work experience has laid a good foundation for the improvement of communication ability, and the more clinical communication with patients, the more convenient communication will be in clinical work (44). Clinical communication skills will naturally become higher and higher. In addition, we found that the more extroverted nursing students are, the better their clinical communication skills are. Previous studies (45, 46) have also confirmed that personality characteristics have an impact on the communication ability of nursing students. The clinical communication ability of nursing students with positive professional attitude, endogenous learning motivation, serving as student cadres and extroverted like to make friends is stronger.

The emotional management ability of nursing students is positively correlated with clinical communication ability. Some studies (47, 48) has indicated that the capacity for emotional management significantly enhances the decision-making and clinical communication skills of healthcare professionals. Furthermore, it fosters a patient-centered work environment that encourages teamwork, thereby exerting a substantial influence on the improvement of patient satisfaction. During clinical practice, when nursing students are confronted with disruptive negative emotions, the application of flexible and effective emotional regulation strategies can mitigate the adverse impact of these emotions. This, in turn, can enhance their clinical communication skills and potentially their overall clinical practice competencies (49). It is essential to prioritize the enhancement of emotional management skills among nursing students, as this plays a crucial role in their overall professional development. By focusing on this aspect, not only can their self-awareness and understanding of others and their environment be enhanced, but also their capacity for effective clinical communication can be significantly boosted. The cultivation of emotional management skills should be an integral part of the training curriculum in nursing education. This involves a multifaceted approach that includes both theoretical learning and practical application (50).

Notably, the current study’s findings may be influenced by a multitude of unmeasured variables that could significantly impact the emotional management and communication skills of nurses. These factors extend beyond the immediate scope of the research and include, but are not limited to, the quality and comprehensiveness of the training programs they undergo. The mentorship and support systems available to these nursing students, which can play a pivotal role in their professional development and confidence, are also crucial. Additionally, the stress levels within the work environment, often characterized by high-stakes situations and patient care demands, can have profound effects on a nurse’s ability to manage emotions and communicate effectively. These elements, while not directly assessed in this study, are integral to a holistic understanding of the factors that contribute to the professional competencies of nurses. Future research endeavors should consider incorporating these variables into their analyses to provide a more nuanced and comprehensive perspective on the development of emotional intelligence and communication skills among nursing professionals.

This investigation acknowledges several limitations. Firstly, the scope of the study was confined to nursing students within our hospital, which restricts the generalizability of the findings due to the limited sample size and inherent data constraints. Secondly, the reliance on self-reported questionnaires can introduce bias, as individuals may overestimate their abilities or give socially desirable responses. Thirdly, the influence of other potential variables on the emotional management and communication skills of nursing students was not fully explored. Fourthly, this survey lacks consideration about the level of training or studying of these nurses, longitudinal tracking of the changes in emotional management and communication skills among nursing students at different stages is of significant importance. Finally, the cultural context of this study is situated in China, a country that exhibits significant cultural disparities from Western societies, which in turn influence communication patterns. Given that this research focuses specifically on a segment of the healthcare community and the conditions of nursing students within educational programs, it underscores the need for additional research reports that encompass a broader range of regions and diverse populations. Such an approach will provide a more comprehensive understanding of the cultural nuances and communication dynamics across different settings.

## Conclusions

In summary, this study has found that the clinical communication and emotion management ability of nursing students are average and need to be improved. Besides, gender, educational level and home places are the independent influencing factors on the emotional management ability in the nursing students. Family structure, internship time and personality are the independent influencing factors for the clinical communication ability in the nursing students. It underscores the urgent need for targeted interventions aimed at addressing the specific challenges and gaps that have been identified within the study. Longitudinal studies are needed to determine how emotional management and communication skills develop over time. Besides, it is imperative that future scholarly endeavors focus on the development and rigorous testing of tailored educational and training programs.

## Data Availability

The original contributions presented in the study are included in the article/supplementary material. Further inquiries can be directed to the corresponding author.
